# A Multidisciplinary Approach to a Complex Fatal Attack Due to a Pack of Maremma Sheepdogs: Is It Always an Accident?

**DOI:** 10.7759/cureus.56911

**Published:** 2024-03-25

**Authors:** Matteo Antonio Sacco, Francesco Maria Galassi, Elena Varotto, Laura Landini, Saverio Gualtieri, Wandamaria Mazzuca, Pietrantonio Ricci, Giuseppe Chiaravalloti, Isabella Aquila

**Affiliations:** 1 Department of Medical and Surgical Sciences, Institute of Legal Medicine, Magna Graecia University of Catanzaro, Catanzaro, ITA; 2 Department of Anthropology, Faculty of Biology and Environmental Protection, University of Lodz, Lodz, POL; 3 College of Humanities, Arts and Social Sciences, Flinders University, Adelaide, AUS; 4 Forensic Anthropology, Forensic Anthropology, Paleopathology and Bioarchaeology (FAPAB) Research Center, Avola, ITA; 5 Nucleo Investigativo, Comando Provinciale dei Carabinieri, Catanzaro, ITA

**Keywords:** autopsy, fatal dog attack, dog bites, dogs, forensic sciences

## Abstract

Deaths due to dog attacks are a worldwide issue. Fatal dog attacks may occur in various environments, including the dog owner’s property. A lot of difficulties emerge when the attack involves a pack of dogs, of different species and sizes. In this case, it becomes much more difficult for forensic investigators to evaluate the event, especially regarding the identification of the dog or dogs that caused the death as well as the identification of the owner and the reconstruction of the dynamics for forensic purposes. In this paper, we analyze a specific case of an attack by a herd of Maremma sheepdogs. A crime scene investigation has been carried out. In the first phase of this case, the victim interacted with dogs that had non-aggressive attitudes. Then, after an escape attempt, she was assaulted by about 20 Maremma dogs of different sizes, leading to multiple injuries all over her body. The greatest difficulty was precisely that of reconstructing the dynamics due to the numerous injuries and dogs involved in the attack. The dynamics were divided into several stages following the analysis of the injuries found on the victim. This case study highlights how the forensic multidisciplinary approach has made it possible to precisely reconstruct the event. The analysis of the dogs' state of malnutrition and their suddenly aggressive attitude towards the victim revealed profiles of responsibility of the owner attributable to improper management and training of the animals to defend their property.

## Introduction

Dogs have always been considered man's best friend and many families today own at least one dog. However, it must be considered that about 1.5% of the world's population is the victim of aggression by the common domestic dog [1]. In the United States, a study found that the death rate from dog attacks was 7.1 per 100 million population per year [2]. In Canada, the average number of human deaths from dog attacks was one or two a year [3]. A similar number of fatalities from dog attacks was reported in Australia [2]. In Spain, a study described 16 cases of fatal dog attacks, with one incident in 2010 resulting in two deaths [2]. In the USA, 49 states reported deaths from dog attacks, with Alaska having the highest death rate [4]. The number of deaths as well as the death rate from dog attacks appear to be increasing [4]. In the literature, risk factors for fatal dog attacks include being male, purebred dogs, pet dogs within the victim's property, and lesions on the head, face, or neck [5-8]. The majority of fatal dog attacks happen on the dog owner's property [4]. It was also found that children under the age of 10 are more likely to be victims of fatal dog attacks than any other age group. Furthermore, the lack of proper containment or restraint of dogs is a significant risk factor [4]. Certain dog breeds, including pit bulls and Rottweilers, have been identified as responsible for a higher percentage of fatal attacks [4-6]. A study conducted between 1979 and 1998 analyzed dog breeds involved in attacks on humans, resulting in human dog bite-related fatalities (DBRF) [6]. The results showed that pit bull-type dogs and Rottweilers were involved in more than half of the reported human deaths caused by dog bites. Also, 25 breeds of dogs have been involved in 238 human deaths in the past 20 years. Out of these, 17% of human deaths concerned restrained dogs on their owners' property; of these, more than half involved unrestrained dogs [6]. Data analysis also showed that of the dog-killing dogs seized by the police, 56% were of the American Staffordshire/pit bull terrier type [7].

The Utrecht University Veterinary Behavior Clinic has a list of diagnostic criteria for "dog-killing aggression" that can distinguish it from ritualized intraspecific aggressive patterns. This difference could be due to owner characteristics or a genetic predisposition to react with aggression in contact with other dogs, or a combination of both [7]. Additionally, police reports indicate that some dogs can attack without warning, and vigorously and rapidly [7].

The investigation of fatal dog attacks from a medico-legal perspective demands a collaborative and comprehensive approach that integrates medical and legal viewpoints [4]. Veterinary pathologists, specialists in behavioural medicine, forensic anthropologists, and odontologists play a critical role in the evaluation of the circumstances surrounding the attack, death scene, and autopsy examinations of both the victim and the dog [4]. To date, despite the numerous papers published in forensic literature, neither protocols nor specific investigation procedures have been defined [8]. Even if the literature offers some insights about bite mark investigations to distinguish animal species when the attack involves a pack of dogs of different species, several difficulties could arise in these cases. In this respect, the literature offers many insights about bite mark investigations to distinguish animal species [8].

In these cases, in fact, it becomes much more difficult to investigate the event for forensic purposes (in this regard a distinction should be made between attacks by domestic animals on the body of the owner who died in the home, for example, by natural death), especially as regards the identification of the dog or dogs that caused the death, as well as the identification of the owner and the reconstruction of the dynamics. We also point out that it is very difficult to assess for judicial purposes whether the event occurred simply by accident or with unintentional or even homicidal purposes. Besides, judicial consequences for the dog and the owners of the dog possibly involved in cases of death may be different. The dog could be transferred or even euthanized. Further, according to the local law of the state where the event occurred, the sentence could be imprisonment for several years. Specifically, in attacks due to packs of dogs, often with multiple overlapping lesions created by dogs of different species, this fundamental reconstruction can prove to be particularly complex. In this work, we propose a complex case of death caused by an attack by a pack of dogs and we illustrate the investigations that have been carried out for the reconstruction of the dynamics. This study has proved to be fundamental in solving the case and assessing the crime during the trial. To this end, we propose an investigation protocol useful for solving these complex cases.

## Case presentation

We describe the case of a 20-year-old woman attacked by a pack of dogs while she was taking a walk in the woods. There was a witness to the event. The dogs belonged to a pasture, so the girl did not know them. The woman was surrounded by dogs. In the first phase, the woman played and interacted with the group made up of about 20 different dogs. In the second phase, upon the arrival of the dominant dog in the group, the girl was suddenly attacked. Rescue arrived when the victim was already dead. The arrival of the police at the scene required the use of firearms to drive away the dogs.

A crime scene investigation has been carried out. In total, 18 Maremma sheepdogs were captured. All wounds on the victim's body were analyzed. A careful collection of dog hair was carried out on the hands and the body. An autopsy was performed. All organs were photographed, examined and fixed in 10% formaldehyde. Biological organs and fluids were taken for toxicological and histopathological analysis. The lesions were photographed and the distances between the pairs of lesions (bite marks) were measured. The autopsy results were evaluated by forensic anthropologists and a forensic zoologist. Post-mortem toxicological investigations were carried out using immunofluorescence and immunochromatography methods.

**Figure 1:**
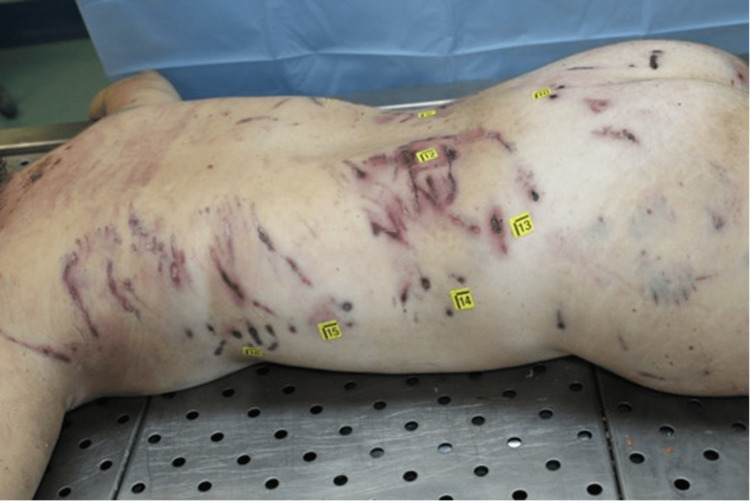
Assessment of bites and scratches on the body

**Figure 2:**
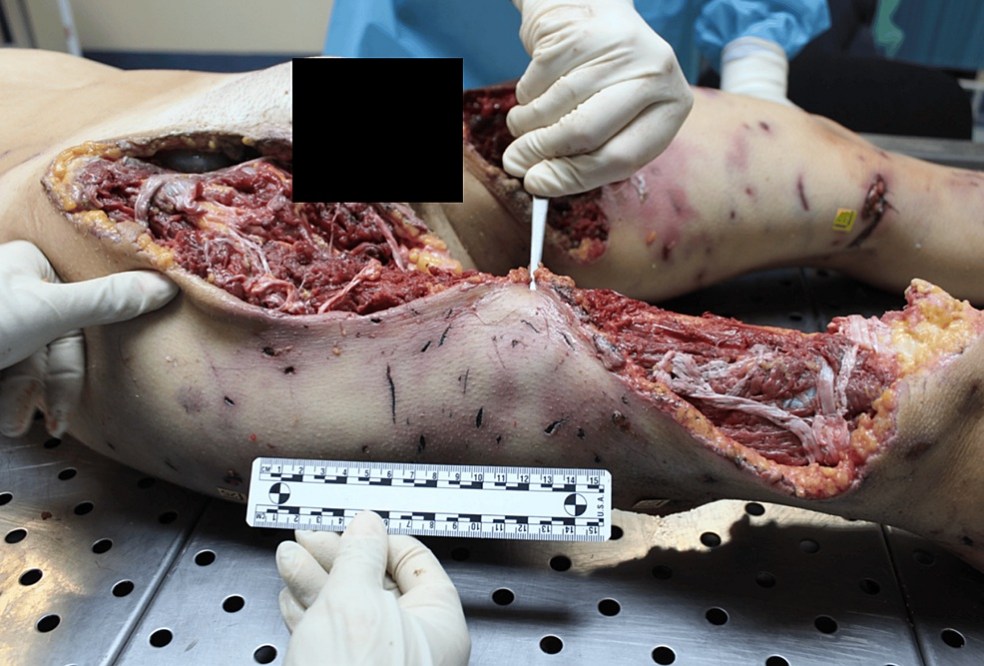
Avulsion injury of right lower limb with numerous bites

**Figure 3:**
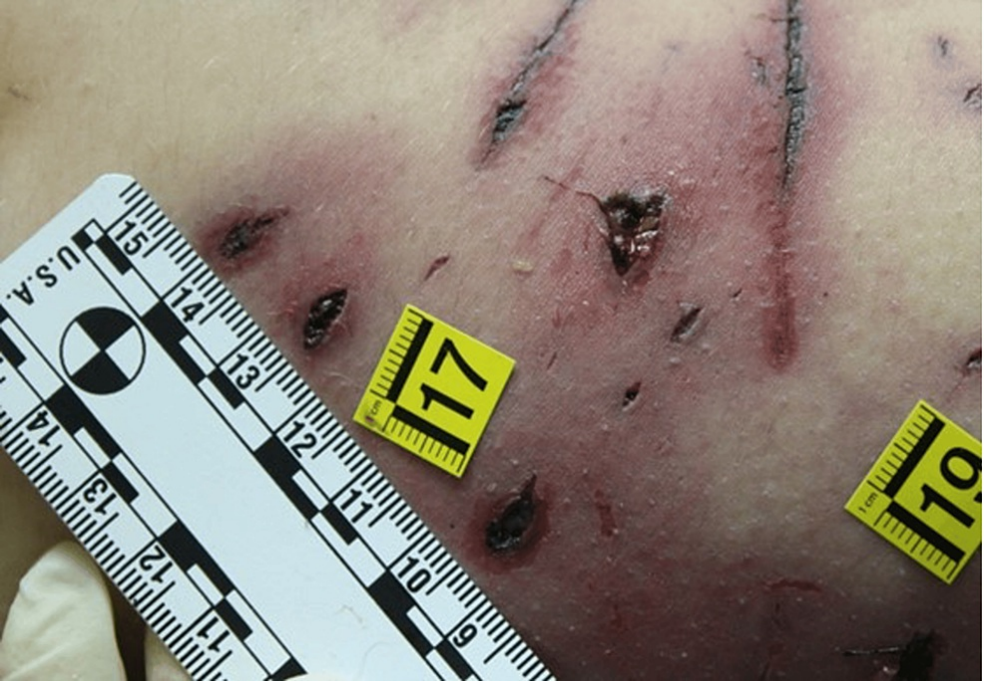
Detail of the morphological aspect of the bites on the lower limb

**Figure 4:**
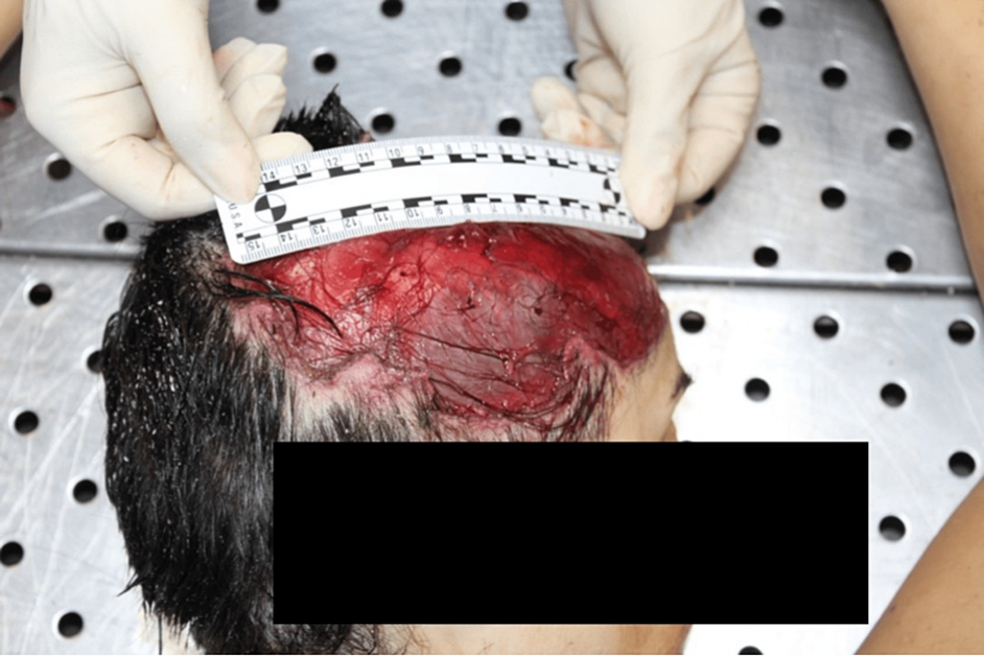
Analysis of the head which appears partially without the scalp

The victim wore only a shirt and bra, which were stained with blood and mud. On external examination, the body exhibited numerous mixed-type injuries to the head, trunk and limbs. Examination of the body revealed 44 mixed-type injuries. Injuries were represented by particular pinpoint holes in the shape of a "V" and skin lesions with irregular and furrowed edges. There were also parallel skin abrasions and superficial linear abrasions consistent with claw marks on the back. On the head, there was a large, lacerated wound with loss of the scalp and exposure of the cranial vault. An autopsy revealed multiple injuries to the arterial vessels of the lower limbs, including femoral arteries. Toxicological analysis was negative. Histological investigations confirmed the vitality of the injuries (Figures 1-4). 

The cause of death was attributed to hemorrhagic shock due to the injury of arterial vessels of the lower limbs consistent with a pack-dog attack. From the analysis of the injuries, it was inferred that the victim was still alive at the time of the attack by the animals (the data were obtained by evaluating the vitality of injuries i.e. the presence of vital infiltration surrounding the injuries. The conclusion was supported by histopathological analysis) and that the depletion, especially of the lower limbs, continued after death. There were defensive injuries on the upper limbs. From the examination of the injuries it was possible to identify the size of the interdental space of the canines, which provided information about the size and number of dogs.

**Figure 5:**
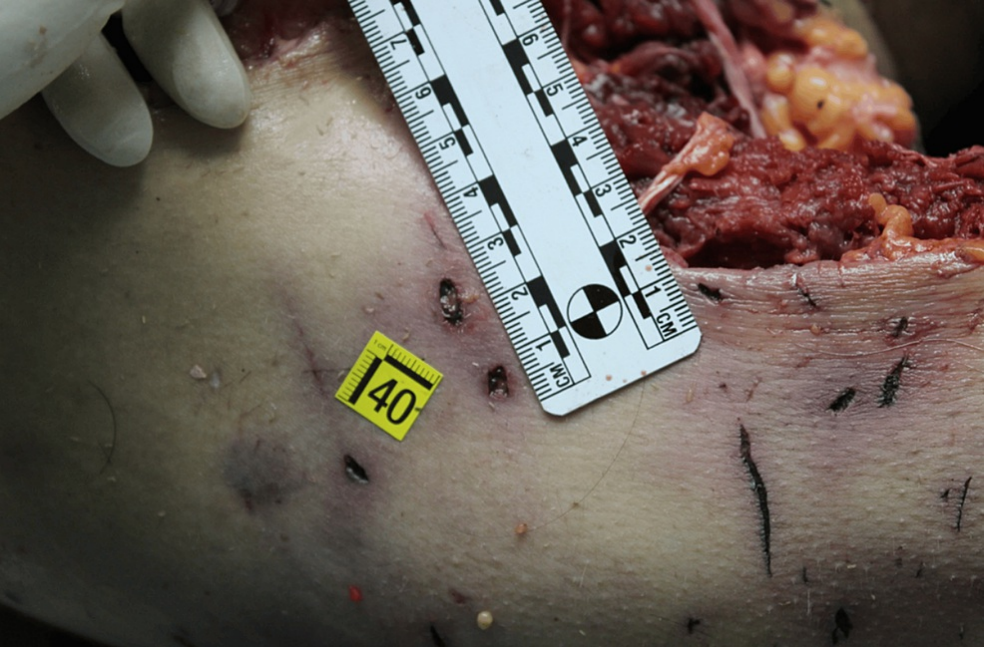
Leg injury due to a small dog

**Figure 6:**
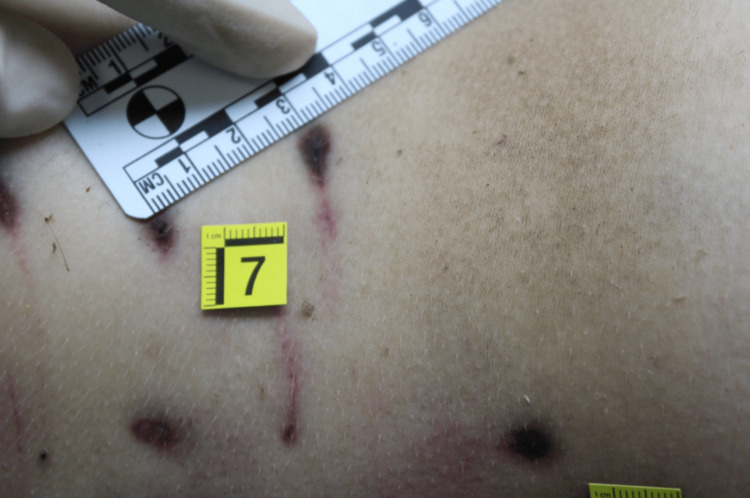
Leg injury due to a medium dog

**Figure 7:**
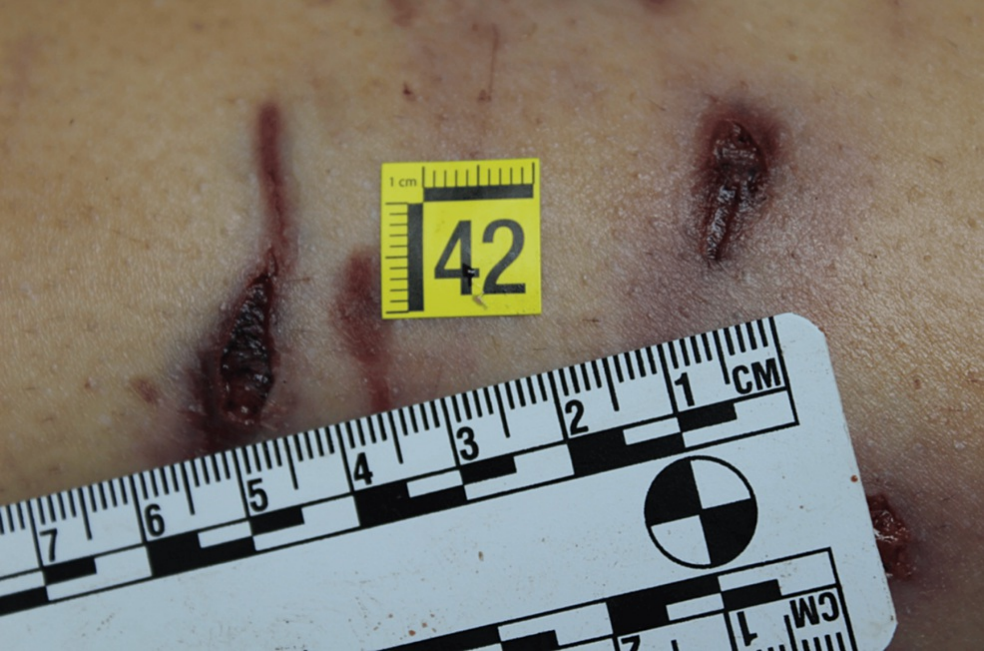
Leg injury due to a big dog

Regarding the number of animals involved it was possible to identify a minimum number of dogs, equal to three. The conclusion was supported as the examination of the previously described lesions made it possible to identify the dimensions of the interdental space of the canines, which provides information on the size of the dogs. In particular, the presence of bites of three different types also provided information on the size of the animals themselves: a small dog, a medium one and a large one (Figures 5-7). 

## Discussion

Dog bites are common occurrences that can cause significant injury. The most common characteristics of dog bites include blunt force trauma due to their powerful jaw closure and teeth with rounded apices, a combination of tearing and compressive forces, lacerations or avulsions of the skin and deeper tissues and puncture wounds [9-10]. Puncture wounds may not be present in some cases due to the rounded apices of their teeth. Furthermore, dog bites often have an "iceberg" feature, with relatively minor skin lesions concealing important lacerations and injuries of deeper tissues and organs [10]. The "hole-and-a-tear" effect is also often present in dog bites. In the case of dog bites, it is common for injuries to occur in the head and neck regions of the victim, and children are particularly vulnerable. Children aged up to six are the most frequently affected by animal bites, of which dog bites are the most common [11], and puncture wounds are found in 83.9% of cases [10]. In addition, fatalities can occur, and research has revealed that different breeds can produce different types of bite marks [11]. The size of the victim and the reason for attacking the victim can affect the distribution of dog bite injuries [10]. The inter-canine distance can help differentiate dog bites from human bites, but there are also other useful bite features like the shape of dental arches, size of the lacerations, morphology, and dogs’ inter-canine width. Further, genetic analysis on biological fluids or animal hair found on the victim can help in the identification of the guilty dogs [6]. Additionally, dog bites can also cause fractures [10], and fatalities are most commonly caused by pit bull-type dogs, Rottweilers, and German Shepherds [12]. Compressive forces may result in depressed skull fractures, chest wall deconstruction, and vascular injury, and even small bites can lead to considerable morbidity [13]. The consequences of dog bites can also be seen in the form of direct structural damage to underlying tendons, nerves, joints, or vasculature [13]. 

Conducting a forensic investigation in a dog attack can be a challenge, particularly when it comes to correctly attributing the injuries [8]. The legal consequences can be different including owner detention and euthanization of the dog. It is important to note that not all dog bites result in fatal attacks; however, dog attacks can cause high-degree injuries [14]. Odontological examination of bite marks and DNA analysis are the most useful methods in confirming dog bites. At the same time, advanced 3D processing and modelling techniques can provide crucial contributions to detecting injuries certainly caused by animal teeth [14]. In addition, the demographics and epidemiological information about the victims, dogs and the scene of the events must be collected [14].

The external examination of the victim's corpse can reveal relevant injuries and biological traces for DNA typing [11]. The investigation involves analyzing victim, animal, and bite-scenario-related factors using a multidisciplinary approach [10]. Moreover, when dogs bite, their saliva may be visible on the human blood surface and can be used for DNA analysis [14]. Forensic investigations involving bite marks and tooth-prints are also used in cases of dog attacks, and canine short tandem repeat (STR) analysis of saliva traces on bite marks can be used to identify the offending dog [14]. Consequently, legal implications may arise in cases of dog bites, particularly in identifying the offending animal [14]. 

Forensic techniques such as STR typing and bite mark analysis are used to identify the specific animal [15-16]. The literature also mentions canine-specific STR profiles obtained from the dog's saliva left on the wound area as a method of identification [16-17]. Bite marks, tooth prints and canine-specific STR profiles are among the forensic techniques used for identification [17]. The goal of the approach is to find the aggressor's genetic profile. DNA analysis can be used as a forensic technique to investigate dog bites and attacks. In these cases, the dogs that attacked may be identified through DNA analysis in combination with necropsy findings [18].

Children, the elderly, and disabled persons have the highest mortality rates in dog attacks. It is worth noting that victims of dog attacks are sometimes found naked, which can mimic a sexual assault rather than a dog mauling. Therefore, an accurate scene analysis is essential in collecting important information for medico-legal investigations of fatal dog attacks, and the identification of the dog which attacked the victim is necessary for legal and medical investigations.

Dog bite marks show a typical "hole and teardrop" appearance and are produced in two stages: the hole reproduces the penetration of the teeth into the skin, followed by the tearing of the tissues by the shaking of the head [12-13]. The study of the crime scene, the analysis of the corpse and the evaluation of the lesions (location, number and type) can clarify the dynamics of the event and the number of animals. Bite wounds sometimes indicate characteristic tooth marks, while claw scratches can provide indications of paw size [12-13].

In this case, it was possible to reconstruct the dynamics of the event in different phases. In the initial phase, the victim tries to escape but is caught up and pushed to the ground. Then the victim was on the ground, in the prone position, and was bitten on the lower limbs; dogs try to turn the body upside down (as evidenced by grazes on the back). Once in the supine position, the victim was bitten in the groin region and lower limbs, with severing of the femoral artery and massive haemorrhage. Finally, the dogs feed on the victim's lower limbs and try to drag the body with partial scalp removal. 

The reported case shows that, in order to address fatal dog attacks, it is necessary to implement a multidisciplinary approach. This involves accurately assessing physical injuries, exploring the circumstances in which the attack occurred, and reducing the risk of future attacks. Additionally, it is important to consider the dog, the child, the environment, parental supervision, and the dog-child interaction, as well as undertaking educational initiatives to inform breeders and owners about the impact of biological inheritance, early-life experiences, and socialization on aggression towards humans in order to foster a safe and secure environment. Moreover, social fearfulness associated with inadequate socialization during puppyhood and a lack of training and play activities can be addressed through the modification of dogs through socialization, training, and breeding [17-18]. Parent-child supervision and interventions are imperative in minimizing the potential danger of hazards. Furthermore, the psychological impact of dog bite injuries for both the child and parent should not be underestimated and requires treatment. Thus, a multidisciplinary approach is needed to effectively address fatal dog attacks, both in terms of prevention and treatment [17-18].

Challenges to adopting a multidisciplinary approach to managing dog attacks include the need for collaboration between medical, veterinary, and public health professionals. This would enable the development of more cohesive and comprehensive educational programs for children, their parents, and dog owners. In addition, more research is needed to learn the most effective method of knowledge acquisition, retention, and resulting behavioural changes for all ages [17-18]. This includes the need for developing educational materials that are age-appropriate and tailored to the target audience. The education should not only provide knowledge on dog safety but also the importance of responsible pet ownership. It should also include information on proper nutrition, exercise, and the proper socialization of dogs. Furthermore, there needs to be an understanding of the role of the family in providing support for both victims of dog bites and their families. Finally, the development of effective strategies for handling dog aggression in both public and private settings is necessary. This would involve creating programs for recognizing and managing aggressive dogs, as well as providing support to pet owners to help them manage their pets' aggressive behaviour. 

This case study highlights how the forensic multidisciplinary approach has made it possible to define the manner of death. The pack of dogs attacked the victim to defend the herd it was supervising although profiles of responsibility emerged related to the malnutrition status of the dogs which increased their aggression as documented by the forensic veterinarian who carried out the study. A correct analysis of the event is important in order to understand whether it was an accidental or intentional event. In this case, although the event was unintentional in nature towards the girl, the aggressive behaviour of the dogs showed a state of captivity in which they were kept by the owner in order to defend his property.

## Conclusions

Thanks to forensic anthropological analysis, the evaluation of the bite marks (wounds left by the canine imprint) has allowed us to identify at least three dog sizes: small, medium, and large. We underline the role of a multidisciplinary forensic approach in fatal attacks by a pack of dogs: this approach requires the involvement of the coroner, the forensic veterinarian, the forensic anthropologist, and genetic experts for the analysis of biological material. This approach can allow not only the identification of the animals responsible for the attack but also the clear and precise reconstruction of the facts, in accordance with investigative and testimonial data, in order to evaluate whether it was an accident or a premeditated event and the owner's responsibilities in such situations. 
